# Myriad Cardiac Manifestation of Hyperhomocysteinemia

**DOI:** 10.14740/cr395w

**Published:** 2015-04-06

**Authors:** Jai Babu, Prashanth Kumar Malkiwodeyar, Shivashankara Tarikere, Nanjappa Manjunath Cholenahalli

**Affiliations:** a Department of Cardiology, SJICS&R, Bangalore, India

**Keywords:** Hyperhomocysteinemia, Layered left ventricular thrombus, Spontaneous dissection of coronary arteries

## Abstract

Homocysteine has been recognized as a risk factor for various cardiovascular manifestations including thrombosis of arterial and venous system, spontaneous dissection involving various vessels in the body including coronaries and aneurysms. Here we report a young gentleman who was diagnosed as stroke in young and found to have dilated cardiomyopathy, with left ventricular dysfunction and hyperhomocysteinemia. Now the patient was presenting with unstable angina and found to have layered left ventricular thrombus on echocardiography and spontaneous coronary artery dissection on angiography. Our patient is being followed up on optimal medical management, as he is asymptomatic with medications.

## Introduction

Homocysteine has been recognized as a risk factor for various cardiac diseases including thrombosis of arterial and venous system, spontaneous dissection involving various vessels in the body including coronaries, aneurysms, and peripheral artery disease [1]. Here we report a 38-year-old gentleman who was diagnosed as stroke in young and to have dilated cardiomyopathy, with left ventricular dysfunction and peripheral artery disease and hyperhomocysteinemia. Now the patient was presenting with unstable angina and found to have layered left ventricular thrombus on echocardiography and spontaneous coronary artery dissection on angiography.

## Case Report

A 38-year-old gentleman was diagnosed as stroke in young; cardiac evaluation revealed dilated cardiomyopathy with biventricular dysfunction in the past. Serum homocysteine was elevated and was diagnosed to have hyperhomocysteinemia.

**Figure 1:**
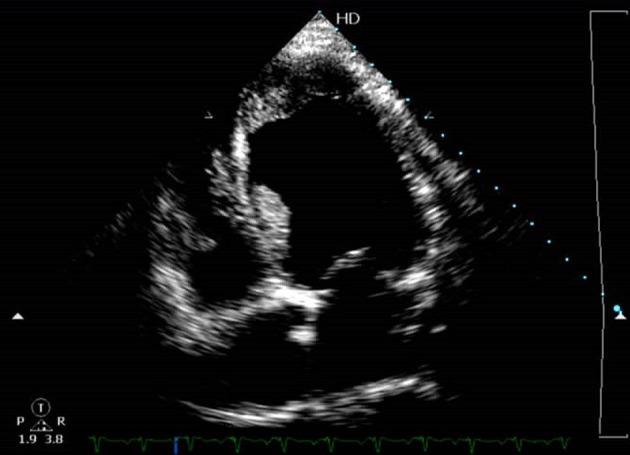
Echocardiography (apical four-chamber view) showing dilated chambers, with layered left ventricular thrombus.

This time he presented with unstable angina, NYHA class III symptoms; patient was non-compliant with medications prescribed earlier. Echocardiography revealed dilated chambers, with layered left ventricular thrombus, and biventricular dysfunction with left ventricular ejection fraction of 22% (Fig. 1).

**Figure 2:**
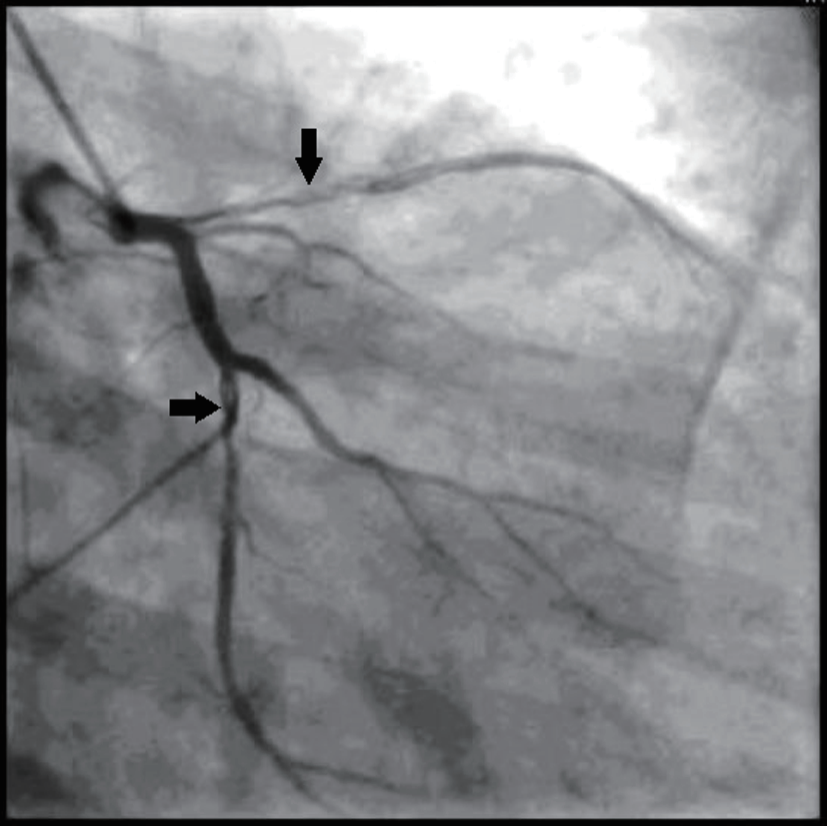
Coronary angiography showing spontaneous dissection in proximal left anterior descending artery and distal left circumflex artery.

Coronary angiography showed spontaneous dissection in proximal left anterior descending artery and distal left circumflex artery (Fig. 2). Patient is being followed up on optimal medical management and regular follow-up.

## Discussion

Hyperhomocysteinemia incidence is estimated to be 5-7% in general population; it is a known risk factor for various vascular disorders which include coronary artery disease, venous and arterial thrombosis, peripheral vascular disease, and cerebrovascular disease [1, 2]. Hyperhomocysteinemia is associated with raised serum levels of homocysteine in general with concentrations more than 14 μmol/L but with no elimination of homocysteine in the urine [3]. Homocysteine is reflected to be noxious to vascular endothelium, leads on to arterial intimal thickening and fibrous rich plaques in smooth muscle cells and collagen relatively than typical fatty plaques noted in atherosclerosis. Further homocysteine promotes endothelial injury, postulated to be facilitated by pro-inflammatory changes, oxidative stress, and endoplasmic reticulum stress, which accordingly lead to endothelial cell injury and dysfunction [4]. Further hyperhomocysteinemia increases thrombotic tendency leading on to further endothelial cell injury, resulting in the early infarctions and death associated with this condition [4].

Hyperhomocysteinemia is associated with arterial and venous thrombus formation. Left ventricular thrombus formation can be seen to be independently associated with hyperhomocysteinemia and left ventricular dysfunction [5]. Hyperhomocysteinemia can even present as left atrial thrombus in sinus rhythm, pedunculated left ventricular mass in different case reports reported in literature [6, 7]. Our patient had layered left ventricular thrombus on echocardiography, which has not been reported in literature to our knowledge. Although biventricular dysfunction with reduced ejection fraction may be an antecedent factor in the cause of thrombus formation, homocysteinemia cannot be ruled out as one of the causative factors in this case.

There have been numerous causes associated with spontaneous coronary artery dissection like atherosclerotic plaque, peripartum period, connective tissue disorders (Marfan’s syndrome), vasculitis, after intense exertion, blunt chest trauma, cocaine abuse, hyperhomocysteinemia and idiopathic [8-12]. Hyperhomocysteinemia is related to spontaneous dissection involving aorta (aortic aneurysm), coronary, intra- and extracranial arteries and peripheral arteries [9, 13-16].

Spontaneous coronary artery dissection in coronary arteries is most frequently located in the left anterior descending coronary artery (45-75%), followed by the right coronary artery (20-33%), left circumflex coronary artery (4-19%) and left main coronary artery involved in < 1%. Left coronary artery dissections are more common in women and right coronary artery dissections occur more frequently in men [9, 10, 17]. Spontaneous coronary artery dissection can present as acute coronary syndrome, unstable angina, non-ST elevation myocardial infarction, ST-elevation myocardial infarction, or chronic stable angina [18], and can even present as refractory congestive heart failure with attempt to bridge to cardiac transplantation [19, 20].

In our case, patient had spontaneous dissection in proximal left anterior descending artery and distal left circumflex artery. This was unique case report in that both spontaneous dissections of coronary arteries present at the time of clinical presentation with unstable angina.

Recognized guidelines are not present to the treatment of patients with spontaneous coronary artery dissection at present. Treatment options include optimal medical management and revascularization with CABG or PCI. Medical management includes antiplatelets, anticoagulation, nitrates, and beta-blockers [21, 22]. Revascularization is contemplated with ongoing ischemia and is frequently treated with percutaneous intervention or surgery and is individualized [9].

Our patient is being followed up on optimal medical management, as he is asymptomatic with medications. He may be considered for myocardial perfusion imaging for inducible ischemia in future for possible coronary revascularization.
